# Geospatial-Temporal and Demand Models for Opioid Admissions, Implications for Policy

**DOI:** 10.3390/jcm8070993

**Published:** 2019-07-08

**Authors:** Lawrence Fulton, Zhijie Dong, F. Benjamin Zhan, Clemens Scott Kruse, Paula Stigler Granados

**Affiliations:** 1 Department of Health Administration, Texas State University, 601 University Drive, San Marcos, TX 78666, USA; 2 School of Engineering, Texas State University, 601 University Drive, San Marcos, TX 78666, USA; 3 Department of Geography, Texas State University, 601 University Drive, San Marcos, TX 78666, USA

**Keywords:** opioids, GIS, random forests

## Abstract

*Background:* As the opioid epidemic continues, understanding the geospatial, temporal, and demand patterns is important for policymakers to assign resources and interdict individual, organization, and country-level bad actors. *Methods:* GIS geospatial-temporal analysis and extreme-gradient boosted random forests evaluate ICD-10 F11 opioid-related admissions and admission rates using geospatial analysis, demand analysis, and explanatory models, respectively. The period of analysis was January 2016 through September 2018. *Results:* The analysis shows existing high opioid admissions in Chicago and New Jersey with emerging areas in Atlanta, Salt Lake City, Phoenix, and Las Vegas. High rates of admission (claims per 10,000 population) exist in the Appalachian area and on the Northeastern seaboard. Explanatory models suggest that hospital overall workload and financial variables might be used for allocating opioid-related treatment funds effectively. Gradient-boosted random forest models accounted for 87.8% of the variability of claims on blinded 20% test data. *Conclusions:* Based on the GIS analysis, opioid admissions appear to have spread geographically, while higher frequency rates are still found in some regions. Interdiction efforts require demand-analysis such as that provided in this study to allocate scarce resources for supply-side and demand-side interdiction: Prevention, treatment, and enforcement.

## 1. Introduction

In the 1990’s pharmaceutical companies began marketing to the medical community that opioids were non-addictive, and medical providers began prescribing them at a higher rate [1]. This marketing opened the door to the U.S. opioid epidemic. Federal funding alone to fight this epidemic was estimated at $7.4 billion in 2018 [2].

The U.S. Department of Health and Human Services estimates that 91,548 people died from opioids, synthetic opioids, and heroine in 2016 [1]. The National Survey on Drug Use and Health estimated that 4.2% of the U.S. population misused opioids in 2017 [3]. The toll in morbidity and mortality is facilitated by over-prescription and bad actors, both individuals and countries.

In April of 2019, 31 physicians, 7 pharmacists, 8 nurse practitioners, and 7 other licensed medical professionals in 7 different states were charged as part of a law enforcement investigation of providing opioid prescriptions for cash or sex. These individuals prescribed more than 32 million pills [4]. In May 2019, a podiatrist was convicted of operating an opioid pill mill [5]. In another example from May 2019, a Virginia doctor was convicted on 861 counts of drug distribution. The oxycodone and oxymorphone that the physician prescribed to a West Virginian patient resulted in her death [6]. As a final example, 162 individuals including doctors were charged for prescribing and distributing opioids in June of 2018 [7]. The problem with bad actors is real and widespread, yet the distribution of opioid and opioid-like products outside of the medical system may be an even larger problem.

China is the largest U.S. source of illicit fentanyl and fentanyl-like substances, and it distributes that product through Canada, Mexico, and directly to the U.S. [8]. The reason for China’s involvement in our markets is that its pharmaceutical system is poorly regulated [9] and that its manufacturers create new and uncontrolled substances to stay ahead of regulators [10]. One estimate is that Chinese fentanyl and derivatives supply 90% of the illicit product in the United States [11]. Even so, Mexico’s two largest criminal organizations traffic the product largely through San Diego. Dominican traffickers supply the heavily stricken northeast [12].

The net result of over-prescription, illicit actors, and illicit suppliers is an increase in morbidity and mortality. Policy considerations for addressing these problems and providing funding for prevention, treatment, and enforcement require an understanding of the geospatial, temporal spread of the epidemic as well as models of demand for services. This research describes the geospatial, temporal spread of opioid inpatient demand and prevalence and provides explanatory models for opioid admissions. Actual met demands might be aggregated to estimate state-level admissions as well as resource requirements. The significance of this research is that it provides decision support for policymakers by identifying areas which require additional enforcement as well as funding. 

## 2. Experimental Section

### 2.1. Data

Data for this research derive primarily from Definitive Healthcare, through the hospital “inpatient diagnosis analytics” query. Only principal diagnoses ICD-10 codes beginning with F11 were used. F11 codes are opioid related disorders. Complete annual data were available for 2016 and 2017; 2018 data were only available through September. The Definitive Healthcare data largely derive from the Centers for Medicare and Medicaid Services (CMS) Standard Analytical Files (SAF) as well as organization estimates all-payor claims through parochial algorithms [13]. The Census Bureau provided population data for rate calculations. [14]

### 2.2. Geospatial Analysis

Heat maps are first used to plot zip-code level claims count data for 2016 through 2018. Subsequently, heat maps illustrate county-level opioid-related inpatient claim per 10,000 population for the same years. For rates, county-level data were selected rather than zip-code level data, as zip-code level data resulted in outliers that influenced interpretation. Some zip codes have very small populations, yet still have admissions. The heat maps illustrate the intensity of opioid admissions and rates by color-coding map areas. When used properly, they can highlight geographic variation [15]. The use of heat maps in healthcare is ubiquitous, as they have been used for improving minority health surveillance [16], examining birth outcomes [17], and many other applications. The value in geospatial-temporal analysis is the graphical depiction of change in demand over time. The significance of changes for 2016 to 2017 claims and claim rates, years with complete data, are evaluated by a non-parametric t-test, the Wilcoxon matched pairs signed rank test, since parametric assumptions such as normality, homogeneity of variance, and independence of do not hold [18]. Matching is performed to account for the geographic unit (zip codes for claims and counties for claim rates).

### 2.3. Explanatory Analysis

Stepwise linear regression, lasso regression, robust regression, elastic net regression, and extreme gradient-boosted random forests estimate the ICD-10 F11 opioid admissions. We built multiple models on an 80% training set to see which one(s) perform best in explaining/forecasting a 20% test set, thus investigating bias-variance trade-off [19]. While regression models provide coefficient estimates for variables, random forests provide the importance of each feature. All models are exploratory to see which workload, financial, technical, and geospatial-temporal features might be explanatory and thus useful for allocation of resources by policymakers.

Stepwise regression models add and subtract variables based on criteria to produce reasonable multiple regression models. In this research, the Akaike Information Criterion (AIC) is used to select the stepwise model using a forward and backwards method. In this method, variables are added in sequence and removed if they no longer contribute significantly to the model’s performance [20].

Lasso regression is a constrained regression that penalizes any model with too many variables using an L1-norm penalty function (absolute value). Ridge regression is similar to lasso regression but penalizes using squared coefficient estimates (the L2-norm). Elastic combines both L1 and L2 penalty functions. Equations (1) through (4) are the parameter estimation models for linear, lasso, ridge, and elastic net regression [20]. The parameter λ in all models is a Lagrangian multiplier, while the parameter α in Equation (4) mixes the squared penalty with the absolute value penalty.

Linear regression (OLS):

(1) $$\hat{\beta }=\overset{N}{\underset{i=1}{\sum }}{\left(y_{i}-\beta_{0}-\overset{p}{\underset{j=1}{\sum }}x_{ij}\beta_{j}\right)}^{2}$$

Lasso regression (L1-norm):

(2) $$\hat{\beta }=\overset{N}{\underset{i=1}{\sum }}{\left(y_{i}-\beta_{0}-\overset{p}{\underset{j=1}{\sum }}x_{ij}\beta_{j}\right)}^{2}+\lambda \overset{p}{\underset{j=1}{\sum }}\left|\beta_{j}\right|$$

Ridge regression (L2-norm):

(3) $$\hat{\beta }=\overset{N}{\underset{i=1}{\sum }}{\left(y_{i}-\beta_{0}-\overset{p}{\underset{j=1}{\sum }}x_{ij}\beta_{j}\right)}^{2}+\lambda \overset{p}{\underset{j=1}{\sum }}\beta_{j}^{2}$$

Elastic net (L1 & L2 Norm):

(4) $$\hat{\beta }=\overset{N}{\underset{i=1}{\sum }}{\left(y_{i}-\beta_{0}-\overset{p}{\underset{j=1}{\sum }}x_{ij}\beta_{j}\right)}^{2}+\lambda \overset{p}{\underset{j=1}{\sum }}\left(\alpha \beta_{j}+\left(1-\alpha \right)\left|\beta_{j}\right|\right)$$

The advantages of regression-type models are that coefficients are easily interpreted. Data, however, need to be scaled, and no one single approach may be best. Regression-type models are unable to find logical dichotomous or polytomous splits in variables that provide explanatory power without researcher specification.

**Figure 1:**
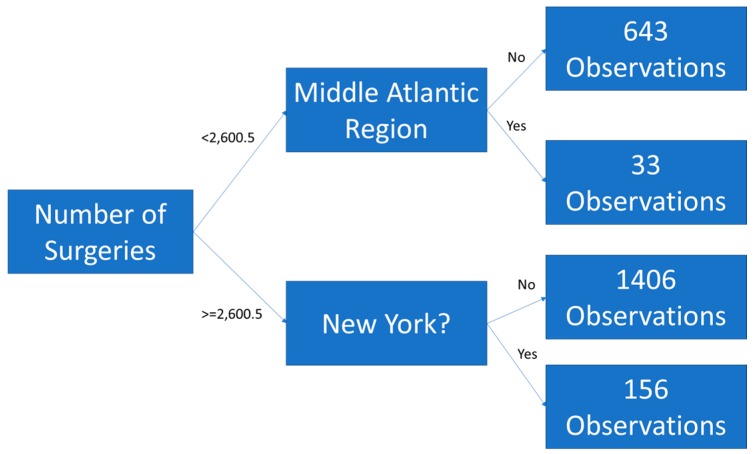
An example of a tree model to classify opioid admissions.

Random forests are a machine learning technique that use an ensemble of de-correlated tree models. The tree forecasts are averaged (ensembled) to produce an estimate. A tree model classifies counts of observations by splitting variables based on some decision criteria. Trees must be pruned or truncated, so that they do not overfit [20]. Figure 1 is an example of a tree built from the Definitive Healthcare F11 dataset with depth of two branches. The tree splits observations by surgeries less than / greater than or equal to 2600.5 then again by region = Middle Atlantic and state equal to New York. The “<0.5” indicates that the region is not the Middle Atlantic and that the state is not New York, as those are dichotomous variables, as estimates for dichotomous variables are not integers in tree models.

Gradient boosted random forests use nonlinear optimization to optimize a cost function based on the (pseudo)-residuals of a function. The residuals of each tree are re-fitted with the possible independent variables in other tree models to estimate a better fit. A more complete discussion of gradient boosting is provided in The Elements of Statistical Learning [20].

The advantages of gradient boosted random forests are that they are scale-invariant, that they find relationships (splits) which the researcher might miss, and that they generate importance metrics allowing researchers to see which variables are explanatory. The disadvantage is that these models will overfit the data if the research does not restrict the growth of the trees. Determining whether a forest is overfit is evaluated by cross-validation (e.g., the test set.)

### 2.4. Variables

All variables derive from the Definitive Healthcare dataset [13]. The primary variable of interest is the inpatient admissions for ICD-10 code F11 (“Opioid-Related Disorders”) which are measured by hospital claims associated with opioids. There are 55 opioid-related codes used in this study (Appendix A). This variable is measured at the hospital level and aggregated by zip code / year for geospatial mapping. The inpatient claims provide a measure of the met demand for services and is suggestive of which areas may need additional funding and resources from health policy decisionmakers.

Independent variable groups evaluated in the explanatory models included financial variables, workload variables, technical variables, and geo-spatial temporal variables. All the variables in the groups are measured at the hospital level by year and reflect opioid and non-opioid contributions. The financial variables are hospital-level and include net patient revenue, net income, cash on hand, assets, and liabilities. Workload variables are also hospital-level and include discharges, emergency room visits, surgeries, and acute bed days. Technical variables include staffed beds, affiliated physicians, employees, percent Medicare or Medicaid patients, ownership status, medical school affiliation, and hospital type. These are also hospital-level variables. Table 1 provides the appropriate definitions and scope of these variables.

Geographic / temporal variables included the Census Bureau region [21] (Table 2), the urban/rural status, the state, and the year. These models will identify characteristics of the facilities providing inpatient care to opioid abusers. As the epidemic spreads or diffuses, these features might be used to anticipate which local facilities are likely to experience an increase in care for these patients. 

### 2.5. Software

All analysis was performed in R Statistical Software [22] and Microsoft Excel 2016 (Microsoft, Seattle, WA, USA). Packages used for the primary analysis in R Statistical Software are cited.

## 3. Results

### 3.1. Descriptive Statistics—Missing Data

Missing data for both ER visits and surgeries for psychiatric hospitals were imputed with zeros. The assumption for this imputation was simply that these values were true zeros (i.e., no ER for some facilities and no psychiatric surgery for others) rather than missing data. While this assumption may not hold, only 3% of the data were missing even if this imputation was not done. After the imputation of missing data with zeros for ER visits and surgeries, a missingness map depicts that only 1% of the data were missing. Because the percent of missing was so small, means were imputed rather than leveraging more sophisticated techniques like multiple imputation. The total number of valid observations for hospital-level data was N = 2090.

### 3.2. Descriptive Statistics—Quantitative Data

Descriptive statistics for the quantitative data are in Table 3. The average number of ICD-10 claims for F11 was 103.47 with a median of 33; however, the standard deviation was 198.19 indicating significant variability. The average reporting hospital had 274 beds, 14,000 discharges, 50,000 ER visits, 10,000 surgeries, 72,000 acute days, 385 affiliated physicians, 2000 employees, and had 42% of the claims paid by Medicare/Medicaid. The average facility had $527,000 in payments for F11, $1.5 million in charges, $403 million net patient revenue, $30.1 million net income, $37 million cash on hand, $524 million in assets, and $213 million in liabilities. On average, a facility was paid about 35% of charges. (Neither payments nor charges were used in models, as they derive directly from claims.)

### 3.3. Descriptive Statistics—Categorical Data

**Figure 2:**
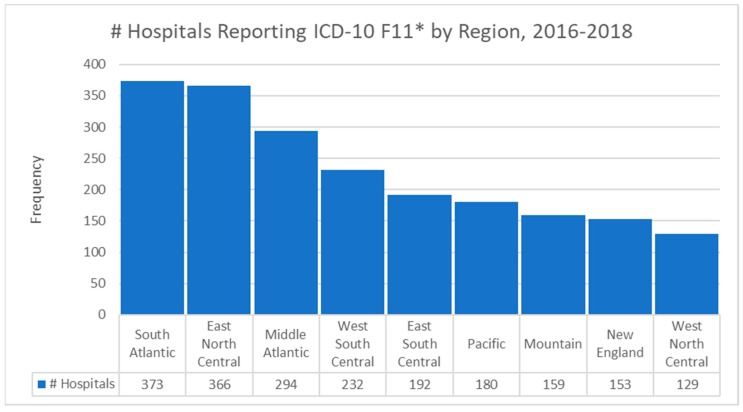
Bar chart of hospital frequencies by Census Bureau region.

The modal Census Bureau region for hospitals reporting F11 claims was the South Atlantic (373, 17.8%) followed closely by the East North Central (366, 17.5%). The bar chart of the hospital frequencies by Census Bureau region is Figure 2. Most of the admissions for F11 codes were in urban areas (1732, 82.9%) with the remainder being areas classified as rural. The South Atlantic region extends from Florida to Washington, D.C., where there is a significant intensity of opioid abuse. The East North Central region includes Chicago, which has extremely high intensity of abuse.

Most of the hospitals reporting these admissions were short-term acute care facilities (1682, 80.5%) with psychiatric hospitals being the next most common type (385, 18.4%). The majority had no affiliation with a medical school (1159, 55.5%), although 21.2% (443) reported a major affiliation. In terms of ownership, 965 (46.2%) were voluntary non-profit non-church owned, 567 (27.1%) were proprietary corporation owned, and 279 (13.3%) were voluntary non-profit church owned. The observations were split nearly evenly between 2016 and 2017 with 1051 and 1039 observations respectively. Most interestingly to policymakers is that the non-profit community of hospitals appears to provide the majority of inpatient care for opioid patients.

### 3.4. Descriptive Statistics—Correlational Analysis

**Figure 3:**
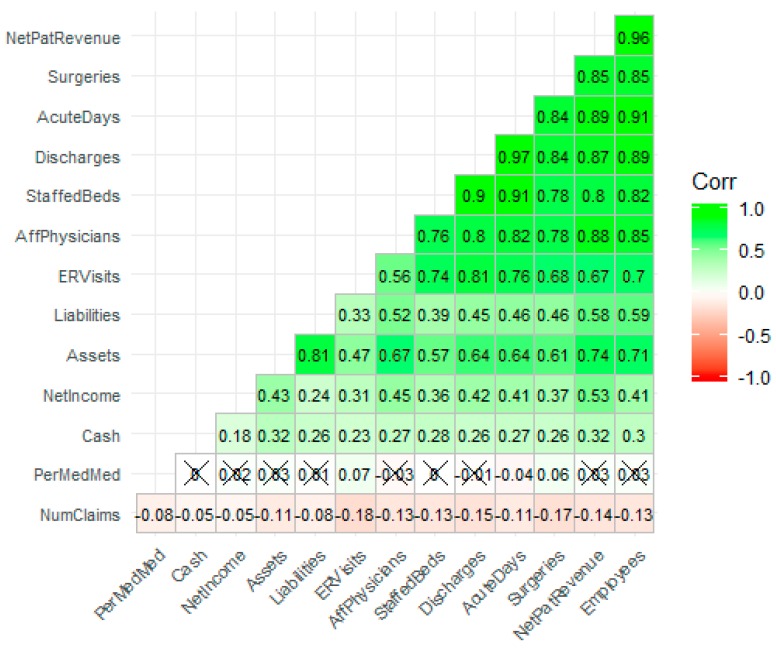
The correlation plot reveals strong relationships among financial and workload variables.

Hierarchical clustered correlational analysis [23], a method which sorts the correlation matrix by the strength of the bivariate associations, revealed strong relationships among most of the workload and financial variables. The strongest correlation is between discharges and acute days (r = 0.97), while the next strongest correlation is between employees and net patient revenue (r = 0.97). All correlations in Figure 3 are statistically significant at the α = 0.05 level unless an “X” appears in the correlation plot. Variable names versus abbreviations are in Table 2. The number of claims appears to be weakly correlated with other variables indicating that the relationships are either nonlinear or not present. What is also interesting from a policy perspective is that as the facility increases in workload and financial metrics, there is a negative relationship with the number of inpatient admissions for F11. This would seem to indicate that smaller hospitals are bearing the brunt of the opioid epidemic for inpatient services. The effect size is small and requires investigation.

### 3.5. Exploratory Data Analysis—Feature Engineering and Transformations

**Figure 4:**
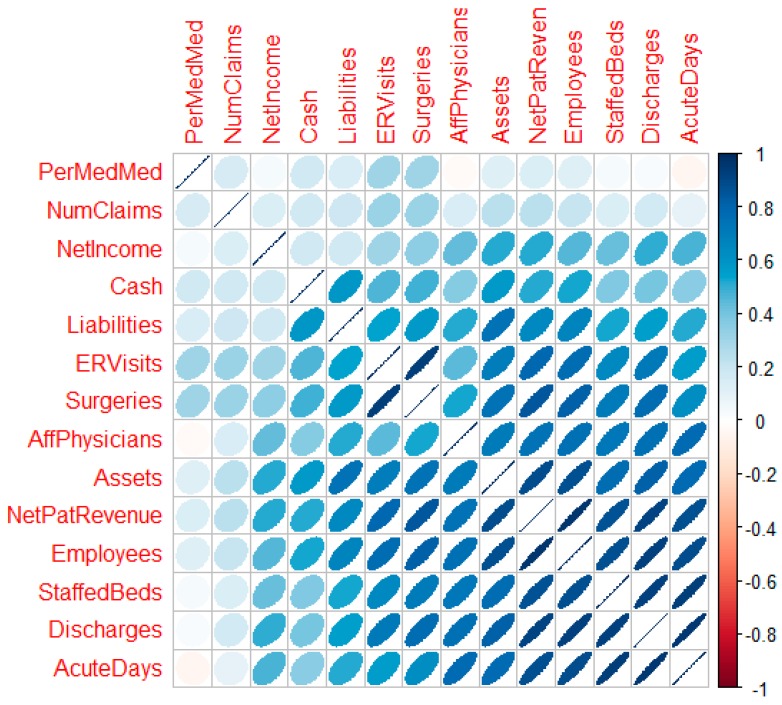
The correlation plot post-transform depicts the bivariate pairs.

Random forest regression models are scale invariant; however, the other methods used in this research are not [20]. The “car” package in R [24] facilitated a multivariate Box-Cox transformation for all modeled quantitative variables simultaneously after adjustment. Box-Cox transformations require that variables be strictly positive definite. With positive definite variables, the transformation seeks power transformations (powers of λ) that make the data multivariate normal enough for use in traditional linear models [25]. This multivariate transformation is particularly useful for random effects models, models where the independent variables are assumed to be not fully observed or the result of random variable draws. The likelihood ratio test of the null (multivariate normal) vs. the alternative (not multivariate normal) after location transformation to make all variables positive definite resulted in a p-value of 1.0. The actual vector of transformations follows: λ = {−0.39, 0.31, 0.38, 0.24, 0.23, 0.34, 0.25, 0.14, 0.1, 0.21, 0.22, 0.48, 0.21, 0.71} for x = {number of claims, number of staffed beds, number of discharges, ER visits, total surgeries, acute days, net patient revenue, net income, cash, assets, liabilities, affiliated physicians, employees, percent Medicare/Medicaid}, respectively. Figure 4 is a correlation plot [26] post-transformation which reveals the strength, direction, and bivariate shape of the bivariate normal between variable pairs. With successful transformation, the forecasting using linear methods is likely to improve. 

### 3.6. Geospatial Analysis Results—Zip Code Unit of Analysis

**Figure 5:**
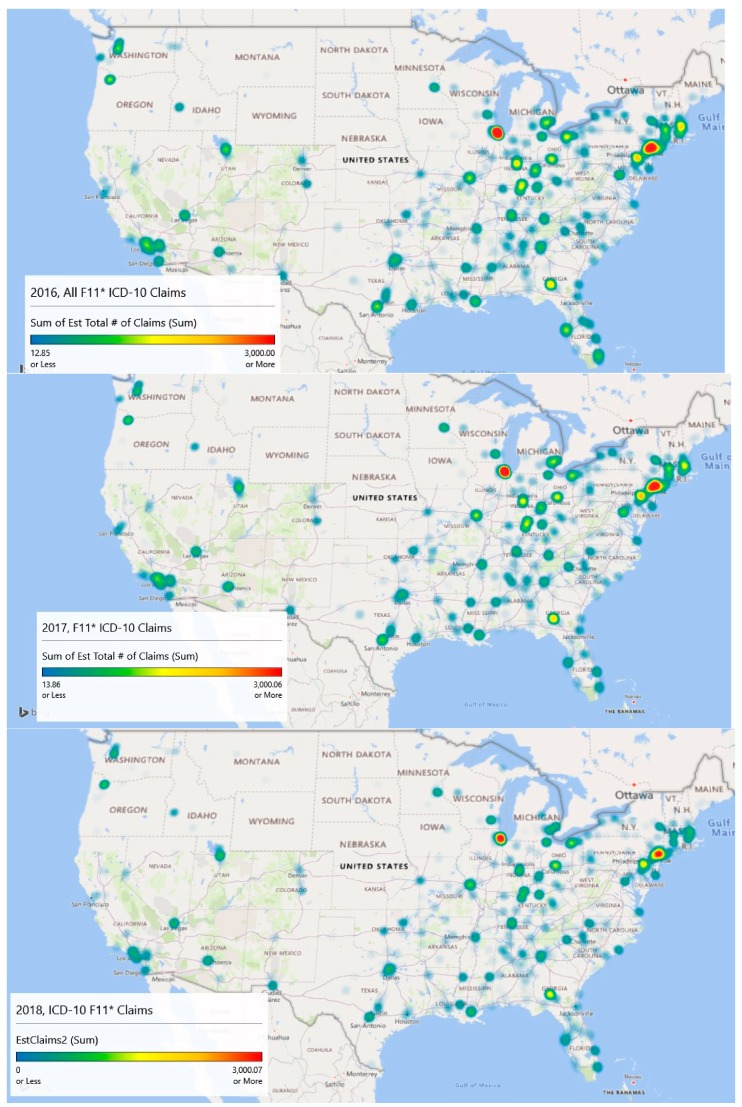
Excel-based heat panel maps of the opioid admissions (ICD-10 F11) for 2016, 2017, and 2018 (extrapolated from September) show emerging areas of interest. Map 1, Map 2, and Map 3 are the **top**, **middle**, and **bottom** maps, respectively.

Geospatial heat map analysis of F11 claims by year and zip code is shown in the Figure 5 panels. These maps were generated by a new feature of Microsoft Excel (3D Map), which links to its Bing mapping service. This study is concerned mostly with supporting resource allocation decision making, so counts of opioid admissions were considered more important than population rates of admissions, although both analyses are provided. With counts, it becomes possible to visualize a proxy for service demand.

The geospatial-temporal heat maps for counts were generated based on scaling to approximately the maximum number of claims experienced in any given year (3000). The meaning of the color ranges is shown in Table 4. The inpatient met demand associated with the opioid epidemic becomes clear with geospatial analysis.

In 2016, the level of intensity for admissions is strongest around Chicago, Illinois and large swaths of New Jersey, where drug overdose is its leading cause of accidental death [27]. The heat map depicts extreme intensity (dark red, near the 100th %) in Chicago. Areas above the median admission rate (yellow) appear to be Washington, DC; Atlanta, GA; and areas of Kentucky, Indiana, and Ohio.

By 2017, the area of intensity around New Jersey had grown, Atlanta saw more intensity, and Chicago remained the most intense. The usage in Los Angeles had expanded but remained sub-intense. Areas in Kentucky, Indiana, and Ohio remained problematic.

Data for 2018 were complete only through September, so they are excluded in the explanatory modeling. However, linear extrapolation produced the 2018 chart which indicates significant intensity in Chicago, New Jersey, and Atlanta. Montana, the Dakotas, Iowa, and Wyoming appear to be inoculated against the epidemic.

Year over year with 2018 extrapolated, there has been a decline in the number of claims. In 2016, the estimated number of claims was 112,816, and that value dropped to 103,436 in 2017. Using linear extrapolation, the estimated number of claims for 2018 is 71,414, although such an extrapolation likely does not reflect later reporting of previous claims and is thus an underestimate. 

A Wilcoxon signed ranks test for complete opioid claims data (2016 and 2017) suggests differences between the years across zip codes with V = 405,660, *p* < 0.001. This result indicates that opioid-related claims from 2017 are statistically lower than 2016 when controlling for the zip code. The approximate 8.3% decrease in 2017 was statistically significant.

Overall, the maps are suggestive of areas where intervention efforts are needed most or are emerging. From a policy perspective, opioid prescriptions in the highest afflicted areas like Illinois and New Jersey might be screened more closely than those (say) in Montana, South Dakota, and North Dakota. Machine learning techniques might be used to identify outliers similar to Ekin et al. [28]. Further, interdiction efforts might focus on Chicago as a major transportation hub along with the emerging problem city, Atlanta, for the same reason, and (of course) New Jersey. 

**Figure 6:**
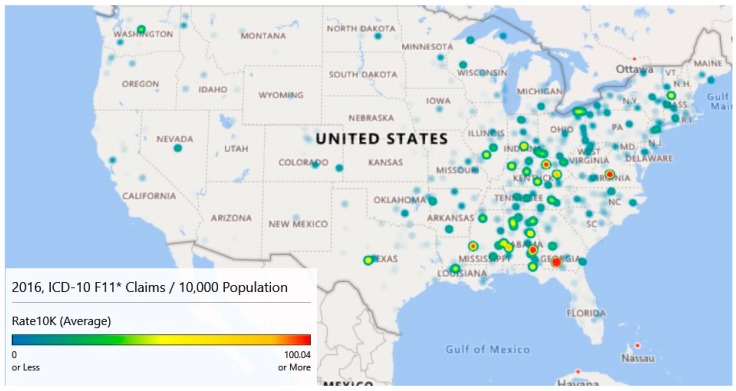

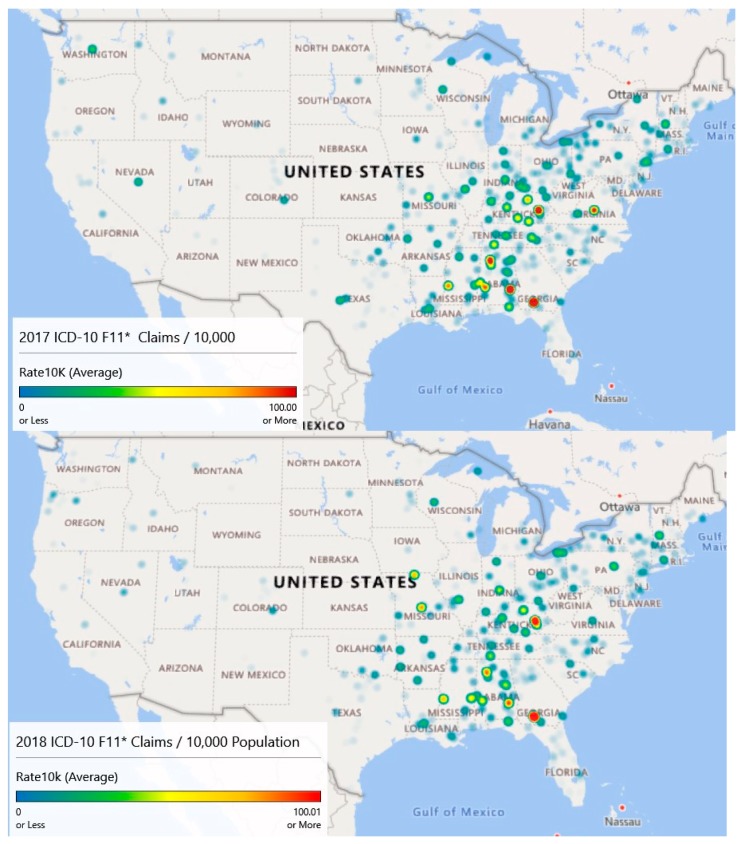
ICD-10 F11* claims per 10,000 population by year shows the Appalachia problem. Map 1, Map 2, and Map 3 are the **top**, **middle**, and **bottom** maps, respectively.

While the counts analyzed and graphed above show areas of interest, rates of claims per 10,000 provide a slightly different descriptive viewpoint. Using county-level population data from the Census Bureau [14], heat maps were generated for 2016–2018. County-level data were used as several zip codes had sparse populations resulting in many outliers. Figure 6 provides the maps for these years. These maps have gradients as specified in Table 4 columns 1 and 2 and are scaled to a maximum of 100+ opioid claims per 10,000 cases for comparison purposes.

Map 1 of Figure 6 (Year 2016) highlights five locations that have intensities of 100/10,000 population or more. The highest intensity claims rate (424.47) is associated with a small county, Colquitt, Georgia. In 2016, this county of 45,492 had an estimated 1,931 claims for a rate of 424.47 per 10,000 population. Norfolk City, Virginia; Bourbon, Kentucky; Bullock, Alabama; and Warren, Mississippi also had claim rates per 10,000 rates. Map 1 also highlights high claim rates in Appalachia.

Map 2 of Figure 6 (Year 2017) illustrates the diffusion or spread of the problem. While counts may have decreased since 2016, intensity appears to have increased, particularly in the Appalachian region and on the Northeastern seaboard. Diffusion is visible as evidenced by areas of intensity that have spread to Missouri.

Finally, map 3 of Figure 6 (Year 2018) is based on extrapolated data, as information was only available through September. Still, the claim rates per 10,000 population for opioid admissions appear to be heaviest in the Appalachian Mountain regions. 

Year over year, the average opioid admissions claim per 10,000 population has declined from 6.1 per 10,000 in 2016 to 5.8 per 10,000 in 2017. The estimate for 2018 is 4.5 per 10,000. While the average rates have declined, there has been noticeable diffusion based on an analysis of the heat maps.

A Wilcoxon signed ranks test for complete opioid claims rate data (2016 and 2017) suggests no differences between the years across counties with V = 109990, *p* < 0.097. This result indicates that opioid-related claims rates from 2017 are not statistically different from 2016 when controlling for the county.

Figure 5 and Figure 6 illustrate different sides of the opioid epidemic problem. Figure 5 provides resource allocation decision-making for treatment, as it illustrates the diffusion across geography. Figure 6 provides decision support for enforcement and prevention, as it shows the higher concentrations of opioid-related admissions. From Figure 5 and associated analysis, we noticed geographic diffusion in terms of counts and that there was a statistically significant difference in the number of claims between 2016 and 2017 after accounting for geography. Figure 6 and its associated analysis shows high claims prevalence rates in Appalachia and that claims prevalence rates year to year remain relatively constant when accounting for geography. Both counts and rates may be useful in supporting resource allocation decision making. 

### 3.7. Explanatory Modeling Results

The first explanatory model, stepwise regression, investigated the number of inpatient opioid claims as a function of the independent variables. Models were built on an 80% training set and applied to 20% blinded test set for analysis of performance. The final stepwise model, the one with the smallest Akaike Information Criterion, included (1) staffed beds, (2) discharges, (3) emergency room visits, (4) surgeries, (5) assets, (6) affiliated physicians, (7) percent Medicare/Medicaid, (8) medical school affiliation, (9) hospital type, (10) year, and (11) state. Unfortunately, this model was only able to account for 17.73% of the dependent variable’s variability. The root mean squared error (RMSE) of the forecast predictions was 1.76. The largest contributions to the model were from the ER visits (Sum of Squares (SS) = 1.49, 1 degree of freedom, df) and from the state (SS = 1.25, 51 df). All variables in the model were statistically significant largely, due to sample size. The overall effect size, however, is small. 

Lasso, ridge, and elastic net regression models built using “glmnet” [29] provided only slightly more variance capture with R^2^ = {17.82%, 17.77%, 17.77%}, respectively. The RMSE’s were 1.75 for all three models. The elastic net selected a lasso model by assigning parameter α = 0. These models produced are essentially equivalent to the stepwise regression analysis.

**Figure 7:**
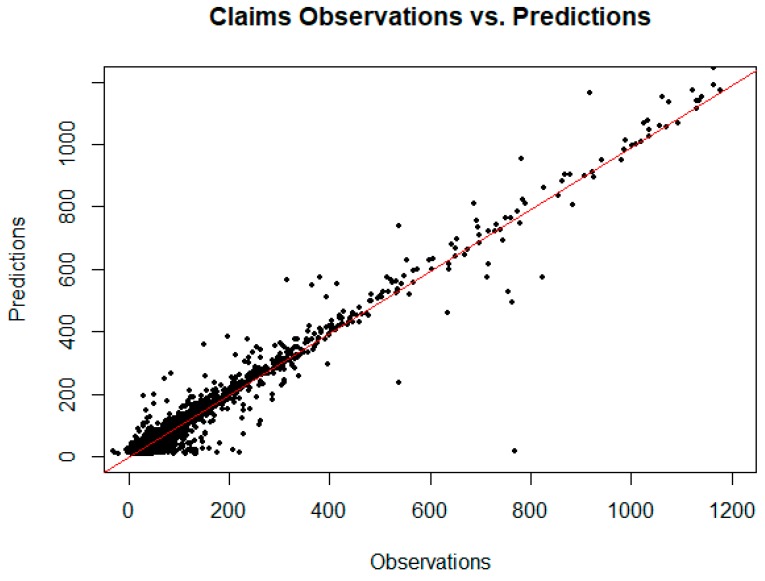
Predictions vs. observations for the claim data based on an extreme gradient boosting random forest provides reasonable predictive accuracy. The R^2^ for the fit on the entirety of the data using the model built only on the training set is 0.965, while the R^2^ for the fit on the test set is 0.878.

Gradient-boosted random forests [30] performed well on the unobserved test set and untransformed data, achieving an R^2^ = 0.878 with hyperparameter tuning (depth of 6 trees, 500 rounds, learning rate of .1). To compare the results more fairly with the regression models, the same random forest configuration was run on the transformed data resulting in R^2^ = 0.550 and an RMSE = 0.06. Figure 7 is a plot of the observed claims versus the random forest predicted claims for the training and unobserved test set data. From this plot, it appears reasonable to forecast demand for opioid inpatient services based on factors important to the random forest model. The implication for policymakers at the state and local level is that resource allocation to treat opioid abuse might reasonably be based on these models.

**Figure 8:**
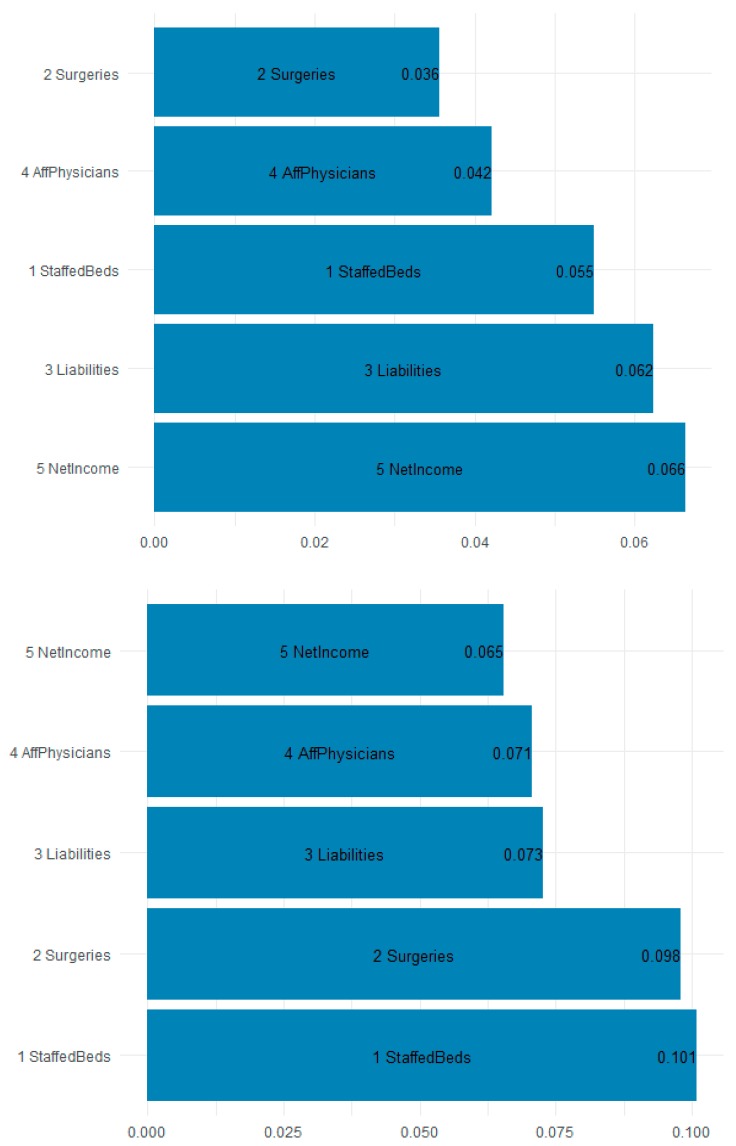
The gain chart (**bottom**) and the cover chart (**top**) show that hospital overall workload and financial variables are explanatory to opioid F11 admissions. Abbreviations are in Table 3.

Figure 8 is a plot of the gain (improvement of an estimate when a feature is used in a tree) and cover (the average proportion of samples affected by splitting using this feature) for the top five items in the importance matrix. The most important features for predicting the F11 opioid claims appear to be the staffed beds (10.1% gain and 5.5% cover), surgeries (9.8% gain and 3.6% cover), and liabilities (7.3% gain and 6.2% cover). Most interesting is that workload and financial variables are the most explanatory. Table 5 shows the top 10 most important features by gain. Because of their predictive accuracy, random forests may be used by policymakers to assign funds and resources to states and localities based on the estimated inpatient demand.

Random forests seek out estimates for each tree to help predict what the demand will be. The splits are not necessarily in the direction one might assume. The purpose of the explanatory models is to assess those workload, financial, technical, geographical-temporal variables that might be useful in estimating which facilities experience demand for ICD-10 opioid admissions. The presence of variables in the tree splits suggest importance only, not directionality. Given that the forest model predicts unseen data with 0.878% accuracy, it seems reasonable to assume that policymakers can rely on the forest for funding allocation determination. The initially explanatory models have outstanding predictive power.

## 4. Discussion

The opioid abuse problem in the United States is non-static. While the U.S. may have seen a decline in prescriptions from 2012 to the present, the average days of supply per prescription has increased [31] and illicit provider activity continues. The contribution of this illicit activity to the problem is likely to intensify the epidemic, which requires no additional assistance. In fact, a March 2018 Centers for Disease Control and Prevention report showed a 35% increase in ER visits for the 16 states most affected by opioids [32]. Policymakers need to consider additional provider controls to ensure that opioids are distributed in accordance with the law.

The GIS mapping of F11 ICD-10 cases through 2018 suggests that the epidemic is diffusing with new growth areas emerging including areas like Salt Lake City, Phoenix, and Las Vegas as evidenced by the orange–red, 75th percentile and above mapping. Policymakers might consider funding prevention, treatment, and interdiction activities according to the GIS trends and demand for inpatient services and might focus analytical techniques to the most highly afflicted cities to target illicit activity by providers.

It is interesting that while California and Florida have large populations, none of their major population centers reached the same level of high intensity scales of other large cities. The questions then become how these patterns might be explained and possibly forecast, and what are the federal and local policy implications for funding based on the expansion/diffusion associated with the epidemic.

The GIS mapping of opioid admission claim rates suggests a higher prevalence rate in Appalachia. This prevalence rate seems fairly constant year over year. Further statistical analysis suggests that, when accounting for geography, there has been no change year over year in the opioid admission claim rates.

The gradient-boosted random forest model was effective in estimating the demand for inpatient services associated with ICD-10 F11. This type of model may be used by policymakers for the allocation of resources and funding to appropriate states, zip codes, or even hospitals. The model suggests that hospital technical and workload factors are important in determining the demand for inpatient services. Specifically, the most important features for predicting the F11 opioid claims appear to be the staffed beds (10.1% gain and 5.5% cover), surgeries (9.8% gain and 3.6% cover), and liabilities (7.3% gain and 6.2% cover). Further analysis of facilities with high demand might be indicative of illicit actors in the community, either individual or otherwise. Such a finding would help prioritize interdiction efforts (enforcement and prevention) and potentially reduce the requirement for treatment; treatment that costs the Federal Government alone $7.4 billion in 2018.

Taken together, the heat map analysis shows diffusion of the opioid epidemic (Figure 5), concentration of the prevalence in Appalachia (Figure 6). Hospital claims prevalence centered in Appalachia match the findings of Figure 6, and the ability to forecast which hospitals are likely to see more inpatient claims through gradient-boosted random forests supports resource allocation decision-making.

## 5. Conclusions

This research is largely descriptive and explanatory in nature, yet it provides some insights about the spread of the opioid epidemic over time and space. In this study, we found that met demand for opioid admissions has concentrations in Chicago, Illinois and large swaths of New Jersey. We also found emerging areas of increased demand in Washington, DC; Atlanta, GA; and areas of Kentucky, Indiana, and Ohio based upon map analysis. Random forest models were able to effectively predict ICD-10 opioid claims with high accuracy (R^2^ = 0.878), and staffed beds, the number of surgeries performed, organizational liabilities, and the number of affiliated providers were the most important features in doing so.

Limitations of this study include the fact that some locations and states (e.g., Texas, Florida, and California) are likely to experience higher admissions, as they may be associated with opioid inpatient treatment destinations. The exact zip code of admission may not reflect the zip code of occurrence. The allocation of federal and state resources for inpatient opioid medical services should still reflect the inpatient demand. Further, allocating resources based on inpatient demand will not capture unmet demand, and some demand may exist in specific areas due to higher bed capacities per capita. In other words, it is likely that there are areas which should have higher inpatient census for opioid abuse but do not have the requisite available bed capacity.

This battle is likely to continue for the near future, and with limited assets, policymakers will have to use techniques like those presented here to allocate resources for supply-side and demand-side interventions (prevention and enforcement). While the research only focused on inpatient admission (exceedingly resource intensive), analogous studies for outpatient visits and deaths might be done. This research team will continue describing, explaining, and forecasting opioid-related incidents. 

## Figures and Tables

**Table 1:** Independent variables for hospitals, all patient-types.

**Financial Variables**	**Defined**	**Measurement**
Net Patient Revenue	Gross Patient Revenue less attributable expenses	Ratio
Net Income	Income less costs, expenses, and taxes	Ratio
Cash on Hand	Cash available to the organization	Ratio
Assets	Company owned	Ratio
Liabilities	Company owes	Ratio
**Workload Variables**	**Defined**	**Measurement**
Discharges	Number of patients discharged from admission	Integer
ER Visits	Number of emergency room visits	Integer
Surgeries	Number of surgeries performed	Integer
Acute Days	Number of acute bed days of hospital	Integer
**Technical Variables**	**Defined**	**Measurement**
Staffed Beds	Number of staffed beds operated by hospital	Integer
Affiliated Physicians	Number of physicians affiliated with hospital	Integer
Employees	Number of direct employees of hospital	Integer
% Medicare /caid Patients	Percent of patients reimbursing through Medicare/caid	Ratio
Ownership	Governmental, Proprietary, Voluntary Non-Profit	Categorical
Medical School Affiliation	None, Limited, Major, Graduate Affiliation	Categorical
Hospital Type	Children, Critical Access, Long-Term, Psychiatric, Rehab, Short-Term	Categorical

**Table 2:** Census Bureau geographic regions.

**Region 1: Northeast**
New England (Connecticut, Maine, Massachusetts, New Hampshire, Rhode Island, Vermont)
Mid-Atlantic (New Jersey, New York, and Pennsylvania)
**Region 2: Midwest**
East North Central (Illinois, Indiana, Michigan, Ohio, Wisconsin)
West North Central (Iowa, Kansas, Minnesota, Missouri, Nebraska, North Dakota, South Dakota)
**Region 3: South**
South Atlantic (Delaware, Florida, Georgia, Maryland, North Carolina, South Carolina, Virginia, Washington D.C., West Virginia)
East South Central (Alabama, Kentucky, Mississippi, Tennessee)
West South Central (Arkansas, Louisiana, Oklahoma, Texas)
**Region 4: West**
Mountain (Arizona, Colorado, Idaho, Montana, Nevada, New Mexico, Utah, Wyoming)
Pacific (Alaska, California, Hawaii, Oregon, Washington)

**Table 3:** Descriptive statistics for the quantitative variables.

N = 2090	Variable Name	Mean	SD	Median	10% Trimmed
Claims	NumClaims	103.47	198.19	33	57.14
Staffed Beds	StaffedBeds	273.53	227.59	214	240.19
Discharges	Discharges	14,039.67	13,572.68	10,306.00	11,934.51
ER Visits	ERVisits	50,642.37	49,459.27	43,564.50	44,229.24
Surgeries	Surgeries	10,113.16	12,165.17	7,142.00	7,992.79
Acute Bed Days	AcuteDays	72,140.06	72,658.04	49,710.00	59,421.91
Physicians	AffPhysicians	384.89	391.09	312	318.58
Employees	Employees	1,968.09	2,458.91	1,222.50	1,475.25
% Medicare/caid	PerMedMed	42.00%	16.00%	42.00%	42.00%
Net Patient Revenue ($1 M)	NetPatRevenue	$403.07	$519.72	$247.83	$298.78
Net Income ($1 M)	NetIncome	$30.25	$109.98	$8.12	$18.94
Cash on Hand ($1 M)	Cash	$36.59	$182.67	$1.35	$10.53
Assets ($1 M)	Assets	$524.23	$961.34	$206.53	$317.09
Liabilities ($1 M)	Liabilities	$212.69	$542.10	$70.82	$125.23

**Table 4:** Color meanings of the heat maps for counts.

Color	Percentile Value	Lower Value	Higher Value
Blue	0%	0	0
Blue Green	(0, 25%)	1	749
Green	25%	750	750
Green Yellow	(25%, 50%)	751	1499
Yellow	50%	1500	1500
Yellow Orange	(50%, 75%)	1501	2249
Orange	75%	2250	2250
Orange Red	(75%, 100%)	2251.00	2999.00
Red	100%	3000.00	3000.00

**Table 5:** This table provides the gain for the top 10 most important features.

Feature	Gain
Staffed Beds	10.09%
Surgeries	9.79%
Liabilities	7.27%
Affiliated Physicians	7.07%
Net Income	6.54%
ER Visits	5.46%
% Medicare/caid	5.32%
Employees	5.20%
Year 2018	5.04%
Illinois	4.35%

## Appendix A

**Table A1:** Opioid-related ICD-10 codes.

F11 Opioid Related Disorders	F11.24 with Opioid-Induced Mood Disorder
F11.1 Opioid abuse	F11.25 Opioid dependence with opioid-induced psychotic disorder
F11.10 …… uncomplicated	F11.250 …… with delusions
F11.11 …… in remission	F11.251 …… with hallucinations
F11.12 Opioid abuse with intoxication	F11.259 …… unspecified
F11.120 …… uncomplicated	F11.28 Opioid dependence with other opioid-induced disorder
F11.121 …… delirium	F11.281 Opioid dependence with opioid-induced sexual dysfunction
F11.122 …… with perceptual disturbance	F11.282 Opioid dependence with opioid-induced sleep disorder
F11.129 …… unspecified	F11.288 Opioid dependence with other opioid-induced disorder
F11.14 …… with opioid-induced mood disorder	F11.29 …… with unspecified opioid-induced disorder
F11.15 Opioid abuse with opioid-induced psychotic disorder	F11.9 Opioid use, unspecified
F11.150 …… with delusions	F11.90 …… uncomplicated
F11.151 …… with hallucinations	F11.92 Opioid use, unspecified with intoxication
F11.159 …… unspecified	F11.920 …… uncomplicated
F11.18 Opioid abuse with other opioid-induced disorder	F11.921 …… delirium
F11.181 Opioid abuse with opioid-induced sexual dysfunction	F11.922 …… with perceptual disturbance
F11.182 Opioid abuse with opioid-induced sleep disorder	F11.929 …… unspecified
F11.188 Opioid abuse with other opioid-induced disorder	F11.93 …… with withdrawal
F11.19 …… with unspecified opioid-induced disorder	F11.94 …… with opioid-induced mood disorder
F11.2 Opioid dependence	F11.95 Opioid use, unspecified with opioid-induced psychotic disorder
F11.20 …… uncomplicated	F11.950 …… with delusions
F11.21 …… in remission	F11.951 …… with hallucinations
F11.22 Opioid dependence with intoxication	F11.959 …… unspecified
F11.220 …… uncomplicated	F11.98 Opioid use, unspecified with other specified opioid-induced disorder
F11.221 …… delirium	F11.981 Opioid use, unspecified with opioid-induced sexual dysfunction
F11.222 …… with perceptual disturbance	F11.982 Opioid use, unspecified with opioid-induced sleep disorder
F11.229 …… unspecified	F11.988 Opioid use, unspecified with other opioid-induced disorder
F11.23 …… with withdrawal	F11.99 …… with unspecified opioid-induced disorder
